# Social support attenuates the link between torture exposure and post-traumatic stress disorder among male and female Syrian refugees in Sweden

**DOI:** 10.1186/s12914-019-0214-6

**Published:** 2019-09-05

**Authors:** Maria Gottvall, Marjan Vaez, Fredrik Saboonchi

**Affiliations:** 1 Department of Health Sciences, The Swedish Red Cross University College, PO Box 1059, SE-141 21 Huddinge, Sweden; 2 Clinical Psychology in Health Care, Department of Women’s and Children’s Health, Uppsala University, Akademiska sjukhuset, SE-751 85, Uppsala, Sweden; 3 Division of Insurance Medicine, Department of Clinical Neuroscience, Karolinska Institutet, SE-171 77, Stockholm, Sweden

**Keywords:** Post-traumatic stress disorders, Torture, Social support, Refugees, Gender, Protective factors

## Abstract

**Background:**

The aim of this study is threefold: (i) to establish the psychometric properties and gender invariance of ENRICHD Social Support Inventory (ESSI), which was used for the first time in the present study in the population of Syrian refugees resettled in Sweden; (ii) to assess whether gender moderates the associations between social support, exposure to torture and PTSD; (iii) to assess whether social support mediates the association between exposure to torture and PTSD, and whether this mediation is in turn moderated by gender.

**Methods:**

Data from a cross-sectional and population-based study of a random sample of Syrian refugees (*n* = 1215) resettled in Sweden 2011˗2013 was analyzed within a Structural Equation Modeling (SEM) framework.

**Results:**

Our results indicate adequate fit and gender invariance for a unidimensional model of ESSI. Exposure to torture was associated with lower social support (*B* = ˗0.22, *p* < 0.01) and with higher odds ratio (OR) for PTSD (OR 2.52, 95% Confidence interval (CI) 1.83˗3.40). Furthermore, higher social support was associated with less likelihood for PTSD (*B* = ˗0.56, *p* < 0.001). Social support partially mediated the effect of torture exposure on PTSD (OR 1.13, 95% bias corrected bootstrap CI 1.06˗1.26). Gender did not moderate this pattern.

**Conclusion:**

The results indicate that social support attenuates the link between torture exposure and PTSD, and may function as a protective factor for PTSD among both torture-exposed refugee men and women.

## Background

Global forced displacement has escalated in recent years and over 68 million people worldwide are currently displaced as a consequence of conflict, persecution, human rights violations, or violence [1]. Sweden is the second largest European recipient of refugees [2] and in 2015, Sweden received 163,000 applications for asylum which amounted to twice as many as the year before [3]. Since 2011, almost 120,000 of the asylum seekers in Sweden originate from Syria [3].

Torture is a grave violation of human rights and is prohibited in international human rights law [4]. As defined by the United Nations (UN), torture is “any act that by which severe pain or suffering, whether physical or mental, is intentionally inflicted on a person … by or at the instigation of or with the consent or acquiescence of a public official or other person acting in an official capacity …” [5]. Torture is practiced in over 140 countries, e.g. Syria and other countries where many refugees originate [6]. Torture destroys a person’s sense of self, identity, and trust in other people and is considered a complex trauma [7]. Reported prevalence of torture exposure varies substantially between refugee groups and across studies, but it appears fairly common that as much as 20˗40% of non-clinical samples of refugee groups report experiences of torture [8]. Experiences of torture is a strong predictor of mental ill health, especially symptoms of Post-traumatic Stress Disorder (PTSD) among refugee- and conflict-affected-populations [9]. Reported prevalence of PTSD also varies between refugee groups and across studies; however, rates ranging from 9˗30% have been found [9, 10]. According to a recent study of Syrian refugees in Sweden, about 30% of the participants reported that they had been exposed to torture. As expected, those who had been exposed to torture were approximately three times more likely to have clinical level symptoms of PTSD [11]. Although torture constitutes a risk factor, not all those exposed develop PTSD, which may indicate that some protective factors may exist. Given the gravity of consequences of torture and the alarming reported prevalence, identifying such protective factors in refugee populations are of immense importance. Increased knowledge about protective factors could contribute to more efficient efforts for providing supportive environments, and to improve treatment strategies for affected refugee populations.

Social support and networks are widely considered important protective factors pertaining to mental and physical health among general population [12, 13]. Although there is a lack of consensus about a unified definition of social support [14], the concept has been defined as “the social resources that persons perceive to be available or that are actually provided to them by nonprofessionals in the context of both formal support groups and informal helping relationships [15]. Social support can assume various forms such as companionship, emotional, informational, and instrumental support, and has been conceptualized as either subjectively perceived, or objectively enacted, i.e. received support [15, 16]. As well as inserting a direct beneficial effect on wellbeing, a *stress-buffering effect* has been ascribed to social support, that is, social support protects people from potentially adverse effects of stressful events by providing e.g. enhanced coping resources [16]. Regarding trauma-related PTSD, social support has been suggested to act as such a protective factor among both male and female war-veterans, as well as among trauma-patients [17, 18]. Conversely, low social support has been identified as a risk-factor for PTSD after trauma [19] among e.g. war-traumatized women [20]. Even though social support may have a stress-buffering effect at the initial stages after trauma, it is also possible that the effect decreases over time due to social support not being accessed as a result of trauma-related avoidance from social contact [21].

Migration and particularly forced migration often disrupts social networks, social relationships, and sources of social support [22, 23] and can be the cause of social isolation in the new country [23]. Low social support appears to be widespread among refugees in Sweden [24, 25]. About 36% of men and 28% of women who have been in Sweden for 3˗9 years report low social support, and the proportion for those who have been in Sweden for 10˗19 years are reported at 31% for men and 25% for women [24]. Furthermore, Swedish born and non-refugee-immigrants report higher social support than refugees [24].

The multitude of different conceptualizations and various forms of social support [14], however, imply that validity of the construct’s measurement bears a crucial impact on the inferences made in regard to the role of social support in health outcomes [15]. That is, social support can be measured in a number of different ways, based on various definitions, and ranging from assessment of a global single measure [15, 26] to detailed assessment of different properties of social networks and interactions [27, 28]. In their review of social support measures, Gottlieb and Bergen [15] recommend ENRICHED Social Support Inventory (ESSI) as an excellent screening instrument for social support due to the short length of the scale, and particularly, its ability to be used in non-western populations. ESSI was originally constructed to provide a single global score of social support that measures availability of the four attributes of social support (emotional, instrumental, informational, and appraisal support) among myocardial infarction patients [26, 29]. The measure has since been used in a large number of studies and in diverse populations ranging from i.e. breast cancer survivors [30] to healthy multi-ethnic samples [31]. There is, however, still a lack of evidence in regard to validity of ESSI for assessment of social support in refugee populations.

It has been reported that women are more at risk of developing PTSD after trauma than men [19, 32, 33] and that one of the explanations for this may be the differences in types of traumatic events reported by men and women [33]. However, women seem to be more vulnerable to PTSD even after the same type of trauma [19]. Thus, it is possible that gender disparities in PTSD related risk factors partially account for the high female ratio of PTSD [19, 32]. A contributing factor for a higher frequency of PTSD among women could be gender discrimination, which potentially affects most women across their life span [34]. In a study among female refugees who had survived torture, gender discrimination was found to indirectly and positively influence the risk of PTSD through cumulative trauma disorder, which in turn, had a direct effect on the risk of developing PTSD [34].

Although torture exposure, PTSD symptoms and low social support are common among refugees, the potential function of social support in the links between torture exposure and PTSD have to our knowledge, not been examined. Furthermore, there is a lack of validated measures for assessment of social support in refugee populations. Identifying potential modifiable protective factors is a crucial necessity for prevention of mental ill health in general and PTSD in particular, and this is of particular importance concerning refugee populations. Furthermore, given that rates of torture exposure, PTSD, and the function of social support may differ between refugee men and women, the role of gender in this context warrants closer examination.

The aim of this study is threefold:

1. To establish the psychometric properties and gender invariance of ENRICHD Social Support Inventory (ESSI), which was used for the first time in the present study in the population of Syrian refugees resettled in Sweden.
2. To assess whether gender moderates the associations between social support, exposure to torture and PTSD.
3. To assess whether social support mediates the association between exposure to torture and PTSD, and whether this mediation is in turn moderated by gender.

## Methods

### Study design and participants

The study has a cross sectional design. A random sample of 4000 individuals, out of a known and completed sample frame containing 9662 women and men, aged 18˗64 years from Syria who were granted permanent residency in Sweden on grounds of asylum between 2011 and 2013 was drawn by Statistics Sweden. The sample frame (N = 9662) was identified through the Total Population Register (TPR), covering every individual that has resided in Sweden on a permanent basis held by Statistics Sweden. A simple random sampling was used which means that every individual has the same probability of being chosen from a complete and known sampling frame. In 2016, postal questionnaires in Arabic were distributed to the random sample (*n* = 4000) by Statistic Sweden. Study population consisted of 1215 women and men (response rate 34%) who answered to the questionnaire. The procedure is described in more detail in Tinghög et al. [11].

### Selected measures

A comprehensive questionnaire including measures of mental health was used [11]. The following selection of measures were used in this present study.

#### PTSD

All 16 items from the section on trauma symptoms (section IV) in the Harvard Trauma Questionnaire (HTQ) [35], corresponding to the formal diagnostic criteria for PTSD in Diagnostic and Statistical Manual of Mental Disorders (DSM-IV), were used to estimate PTSD. The 16 trauma symptom items include e.g. intrusive recollection, avoidance, and hypervigilance and each item have four categorical responses scored as 1 to 4 respectively. Higher score indicates more symptom severity. Cronbach’s alpha was 0.92. A mean item score ≥ 2.06, based on previously established value of PTSD among primary care patients in Bosnia Herzegovina [36], was used to make a distinction between PTSD cases and non-cases. In order to be included in the analyses, participants needed to answer 14 or more of the 16 items on the HTQ scale.

#### Exposure to torture

The Refugee Trauma History Checklist (RTHC) [37] was used to assess whether respondents have experienced torture before or during their flight respectively. In addition to torture experience, the checklist includes questions on whether the respondents have experienced seven other potentially traumatic events e.g. war at close quarters and violence, either before or during the flight. The checklist consists of 16 items in total, all answered on a binary outcome scale (Yes/No). The items assessing torture exposure was selected for this study.

#### Social support

To measure Social support, ENRICHD Social Support Inventory (ESSI) was used. ESSI is a short, seven item, self-administered instrument that provides a single score of social support covering different types of support – structural, instrumental, and emotional [15]. On six of the items, the response is rated on a five-point Likert scale that ranges from N*one of the time* to *All of the time.* The last item *Are you currently married or living with a partner?* targets the respondent’s civil status and is answered with *Yes* or *No*. Higher score indicate more social support. ESSI displayed high internal consistency indicated by Cronbach’s alpha at 0.906.

#### Covariate

Age in years was included as a continuous control variable in the analyses.

### Statistical analysis

All the analyses were conducted within a Structural Equation Modeling (SEM) framework. The first step of SEM is to evaluate the measurement model of the included latent variables in a model [38]. The second step consists of evaluation of the overall structural model comprising of covariance and causal links between the included variables. Accordingly, in the present analysis, the measurement model and gender invariance of ESSI was first examined. This was followed by the evaluation of an overall moderated mediation model [39] in which gender was treated as a moderator, social support as mediator, torture exposure as exogenous (predictor) and PTSD as endogenous (outcome) variables. Both torture exposure and PTSD were treated as observed variables due to the assessment methods implied in RTHC and the calculation of cut-off values used in HTQ [36].

#### The measurement model of ESSI

Since ESSI has not previously been used in Syrian refugee population, the measurement model (unidimensional factorial structure) was initially examined by means of confirmatory factor analysis (CFA) [40] with maximum likelihood estimation and robust standard errors (MLR). The overall fit of CFA was assessed using the Satorra–Bentler scaled chi-square test statistics, Comparative Fit Index (CFI), root mean squared error of approximation (RMSEA), and standardized root mean square residual (SRMR). CFI ranges from 0 to 1 with cut-off values of 0.90 for adequacy and a more stringent value of 0.95 for goodness of fit. The combination of fit indices minimizes the risk of rejection of well-fitting models, which sometimes is associated with chi-square test statistics’ sensitiveness to large sample sizes. The included model in the CFA consisted of a unidimensional model [15] of ESSI based on the scale’s 6 items with Likert-type response scale. The binary item targeting civil status was used as a formative indicator [41] in all proceeding analysis. Modification Indices (MI) were inspected to examine theoretically justifiable modifications to improve the fit. Nested models were compared by the Satorra–Bentler scaling correction likelihood ratio (∆*χ*2) test.

#### Invariance of ESSI across gender

Further analyses were performed to establish the invariance of the measurement model of ESSI in women and men. Invariance testing allows valid inferences made on differences on latent variables across diverse population groups [42, 43]. Invariance of measurement instruments can be established along a set of hierarchical order models from configural (invariant factorial structures across groups), metric (adding invariant factor loadings), scalar (adding invariant item intercepts), to strict (adding invariant residuals) invariance [42]. As the increasing equality constrains imply nestedness, the fit of the models was compared by the Satorra–Bentler scaling correction likelihood ratio (∆*χ*2) test. Strict invariance which requires equality of all residuals is seldom achieved [44], thus, conditions of strong or partial scalar invariance (i.e. equality of factorial structure, factor loadings, and several intercepts) were deemed as adequate for further analyses [43].

#### Moderated mediation model of gender and social support

**Fig. 1:**
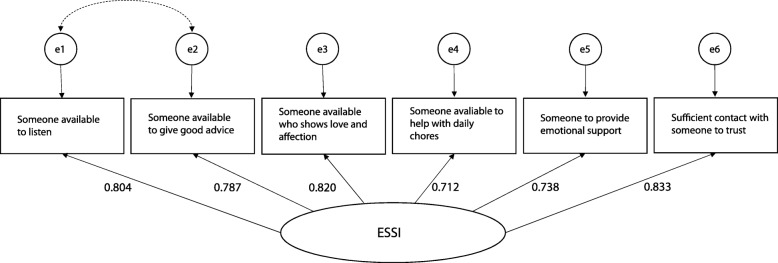
Modified unidimensional model of ESSI

To address the second aim of the study, a moderated mediation model was examined in which gender constituted the moderator, and social support was the mediator in the association between torture exposure and PTSD (see Fig. 1). This hypothesized moderation was examined through multi-group SEM analysis with gender constituting the grouping variable. A multi-group approach to moderation analysis in SEM framework allows testing the hypotheses that single or multiple individual paths within a model are equal across different groups. Moderation is indicated if the best fitting model contains structural paths that differ across the grouping variable. Multi-group analyses with binary outcomes as in the present case is implemented in Mplus 8 through Mixture Model Analysis with known class membership [45]. MLR estimation was used as it provides Odds Ratios (OR) for the individual paths/structural weights containing binary outcomes within the model. Age was grand mean centered and was controlled by being included as a covariate in all the models.

#### Moderation models

First, a reference model when all paths freely estimated (allowing to vary) across gender was specified. According to such a model, all association between the included variables differ across gender. The fit of this model was then compared with a set of models in which equality constraints were placed on structural weights from Torture exposure to PTSD, Torture exposure to Social support, and from Social support to PTSD, to examine potential moderation across different structural weights. First, the paths from Torture exposure to Social support (Model 1) and from Social support to PTSD (Model 2) were held equal individually. Then, both of these paths were held equal across gender simultaneously (Model 3). Finally, all three paths were held equal (Model 4). The increasing number of equality constraints results in nested models with higher degrees of freedom (df). The models were then compared by chi-square difference test based on loglikelihood values and scaling correction factors obtained with the MLR estimator [45], as well as Bayesian information criterion (BIC) to examine moderation.

#### Mediating function of social support

Upon establishing the best fitting multi-group mixture model, mediation analyses were performed by estimating the direct and indirect effects of torture exposure on PTSD with social support treated as mediator using Maximum Likelihood (ML) estimator with 95% bias corrected bootstrap (1000 resampling) confidence intervals (CI).

#### Sensitivity analyses

Due to negative cognitions associated with PTSD, which may result in withdrawal behaviors [21] and may influence social support, sensitivity analyses were performed by examining an alternative structural model in which social support was treated as outcome and PTSD as the mediator. This model was then compared with previous analyses using BIC. Furthermore, to assess the potential impact of non-response rate on the results a set of sensitivity analyses were conducted, first by including the exposure and mediating and moderation variables as well as the outcomes in logistic regression models with both weighted and unweighted data sets, and second, by comparing the results of the main mixture model with weighted and unweighted data.

## Results

### Characteristics of sample

In total, 37.2% women and 62.8% men returned the completed questionnaire (Table 1). The respondents were between 18 and 64 years old (M = 39, SD= 11.34).

**Table 1:** Sociodemographic characteristics of the respondents (*n* = 1215) supplemented with non-response analysis

	*n* (%)	Women/Men *n*	Non-respondents *n* (%)	Respondents vs. non-respondents χ^2^ (*P*-values)
Gender				0.4 (0.52)
Women	452 (37.2)		1008 (36.2)	
Men	763 (62.8)		1777 (63.8)	
Age-groups				68.7 (< 0.01)
18–29	283 (23.3)	122/161	947 (34.0)	
30–39	400 (32.9)	143/257	948 (34.0)	
40–49	295 (24.3)	102/193	545 (19.6)	
50–64	237 (19.5)	85/152	348 (12.5)	
Civil status				
Living with partner/husband/wife	780 (64.9)	315/465		
Level of education				47.2 (< 0.01)
0–9 years	489 (40.2)	176/313	1366 (49.1)	
> 9 years without a university degree	255 (21.0)	86/169	637 (22.9)	
> 12 years with a university degree	471 (38.8)	190/281	790 (28.4)	
Years since immigration				34.0 (< 0.01)
Five or more	79 (6.5)	22/57	324 (11.6)	
Four	334 (27.5)	117/217	845 (30.4)	
Three	802 (66.0)	313/489	1615 (58.0)	
PTSD	353 (30.6)	141/212		
Torture exposure	354 (30.6)	90/264		
Mean score ESSI (SD)		3.5 (1.1) /3.3 (1.1)		

### Confirmatory factor analysis of ESSI

The fit indices for the measurement models of ESSI is presented in Table 2, panel *a*. The unidimensional structure of ESSI displayed a highly significant Satorra–Bentler scaled chi-square (_S-B_χ2 = 119.15; df = 9 *p* < 0.001) and a value of RMSEA greater than 0.05 (0.101, 90% CI = 0.850–0.117), although other fit indices (CFI = 0.961, SRMR = 0.026) indicated approximation to adequacy of the model fit. Examination of MIs suggested that model fit could be enhanced by allowing the error terms of item 1 (*Someone available to listen*) and item 2 (*Someone available to give good advice*) to covary. The modification was deemed theoretically justified due to overlapping item content. The modified unidimensional model of ESSI showed overall excellent fit indices (CFI = 0.991, SRMR = 0.014, RMSEA = 0.052, 90% CI = 0.035–0.071), despite an improved, although still significant, Satorra–Bentler scaled chi-square (_S-B_χ2 = 34.46; df = 8 *p* < 0.001). Satorra–Bentler scaling correction likelihood ratio also showed significant improvement of the model fit with the error terms covariance being estimated compared to the initial model (∆_S-B_χ2 = 72.63, df = 1, *p* < 0.001). The selected modified unidimensional model of ESSI is shown in Fig. 1.

**Table 2:** Fit indices (panel a) and model comparisons for gender invariance of ESSI (panel b)

Model	_S-B_χ^2^	df	CFI	RMSEA (90% CI)	SRMR	Δ _S-B_χ^2^	Δdf	*p*
Panel *a:* Model fit indices								
Unidimensional	119.15***	9	.961	.101 (.085–.117)	.026			
Modified uni-dimensional	34.46***	**8**	**.991**	**.052 (.035–.071)**	**.014**	**72.63**	**1**	**<.001**
Panel *b:* Invariance across gender								
Configural	39.89***	16	.992	.050 (.031–.069)	.015			
Metric vs. Configural	48.62***	21	.990	.047 (.030–.064)	.026	7.445	5	.19
Scalar vs. Metric	63.66***	26	.987	.049 (.034–.064)	.025	15.612	5	.008
Partial scalar vs. Metric	51.482**	**25**	**.991**	**.042 (.025–.058)**	**.027**	**1.49**	**4**	**.83**

** < 0.01 *** < 0.001

Notes: _*S-B*_*χ*^*2*^ Satorra-Bentler scaled Chi-square, *CFI* Comparative Fit Index, *RMSEA* Root Mean Squared Error of Approximation, *CI* Confidence Interval, *SRMR* Standardized Root Mean Square Residual. *Δdf* difference in degrees of freedom. Models in bold are the selected

### Invariance of ESSI across gender

The fit indices for the increasing degrees of invariance of ESSI across gender are displayed in Table 2, panel *b*. Configural and metric invariance were fully established. The scalar invariance, however, showed worsened fit compared to metric invariance (*p* < 0.008). Examining the individual item intercepts revealed that allowing the intercept of item 2 (*Someone available to give good advice*) to vary across gender resulted in partial scalar invariance as all other intercepts could be constrained to be invariant and the model fit did not worsen significantly (∆_S-B_χ2 = 1.49, df = 4, *p* = 0.83).

### Gender as moderator in the associations between exposure to torture, social support and PTSD

**Fig. 2:**
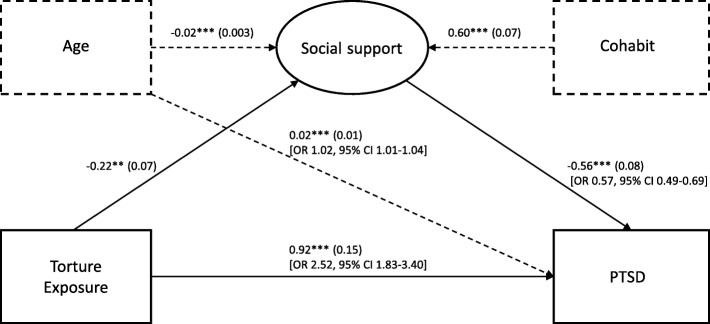
Structural equation model of exposure to torture, PTSD and social support as a mediator. The structural weights are invariant across gender. The displayed estimates are unstandardized coefficients (*B*). The values in parenthesis denote robust standard error. **p < 0.01, ***p < 0.001. Odds ratio and 95% Confidence Intervals are shown in square brackets

Figure 2 displays the structural model of torture exposure, social support, and PTSD for both men and women, with age and cohabitant status included as covariates. In order to examine whether gender moderated this structural model, equality constrains across gender were placed on different paths. Table 3 outlines the comparisons of the structural models with these equality constrains with a reference model within which all paths/structural weights were allowed to differ across gender. As can be seen, none of the models containing equality constrains displayed significantly worse fit compared to that of the reference model (all *p* > 0.05). The most constrained model with all paths forced to be equal across gender (Model 4) was, consequently, selected as the best fitting model as the Satorra–Bentler scaling correction likelihood ratio did not show a significant change from the reference model (∆_S-B_χ2 = 1.22, df = 3, *p* = 0.748). This model also displayed the smallest BIC value (21,907.02), further corroborating the selection. Consequently, gender moderation could not be established and was discarded.

**Table 3:** Comparison of mixture models of torture exposure, social support and PTSD with equality constraints across gender placed on different structural paths within the models

Model (paths constrained to be equal)	BIC	Log likelihood	_S-B_χ^2^	Δdf	*p*
*Reference model* (All paths vary)	21,926.904	˗10819.103	–	–	–
*Model 1* (Torture to social support equal)	21,920.738	˗10819.548	0.810	1	
*Model 2* (Social support to PTSD equal)	21,919.870	˗10819.107	0.008	1	0.927
*Model 3(*Torture to social support, and social support to PTSD equal)	21,913.670	˗10819.527	0.824	2	0.662
Model 4 (All paths equal)	**21,907.020**	**˗10819.723**	**1.220**	**3**	**0.748**

Notes: *BIC* Bayesian Information Criterion, _*S-B*_*χ*^*2*^ Satorra-Bentler scaled Chi-square, *Δdf* difference in degrees of freedom. Model in bold is selected

According to the selected model, those who were exposed to torture were more than two and a half times more likely to suffer from PTSD than the non-exposed participants (OR 2.52, 95% CI 1.83–3.40). Those with higher social support were, on the other hand, less likely to suffer from PTSD than those reporting lower social support (OR 0.57 for a unit change in ESSI, 95% CI 0.49˗0.69). Exposure to torture was associated with lower social support (*B* = ˗0.22, *p* < 0.01). Age as a covariate in the model was negatively associated with social support indicating that older participants reported lower degrees of social support (*B* = ˗0.02 for each year of increased age, *p* < 0.001). The older participants were also slightly more likely to be among those who had PTSD (OR 1.02 for each year of increased age, 95% CI 1.01˗1.04).

### Social support as mediator of the association between the torture exposure and PTSD

Mediation analysis revealed that the total effect of torture exposure on PTSD (2.86, 95% bias corrected bootstrap CI 2.12˗3.93) could be decomposed into a direct effect (OR 2.52, 95% bias corrected bootstrap CI 1.83˗3.40) and an indirect effect mediated by social support (OR 1.13, 95% bias corrected bootstrap CI 1.06˗1.26). Accordingly, social support partially mediated the effect of torture exposure on PTSD. This pattern of mediation did not significantly differ across gender (Wald = 0.821, df = 1, *p* = 0.365), i.e. gender did not moderate the mediation function of social support.

### Sensitivity analysis

The model in which social support was treated as an outcome with PTSD as a mediator showed worse model fit (BIC = 21,924.815) compared to that of the selected model with social support as a mediator (BIC = 21,907.020). Furthermore, in regard to comparison between analyses with weighted and unweighted data sets, neither the point estimates of the associations included in the model nor the significance level of the estimates were altered by using weighted data.

## Discussion

The main purpose of the present study was to assess whether post-resettlement social support among Syrian refugees acts as a potential mediator in the linkage between torture exposure and PTSD, and thus, to examine the indication for social support as a protective factor that may attenuate the association between such a grave trauma exposure and its mental health consequences. Furthermore, given gender differences in social support [46, 47] and its varying associations with mental distress and psychopathology [48, 49], we sought to examine whether such a potential mediating mechanism would differ between refugee men and women. As a first step for pursuing the analyses required to approach these aims, we also established the psychometric soundness of self-report data on social support gathered by ESSI in the study’s population.

Our analyses of the construct validity of ESSI indicate adequate psychometric properties of this measure for use in the Syrian refugee population. The analysis targets the six Likert-scale items of ESSI to handle the inconsistency in measurement levels. It should be acknowledged that there is an ongoing debate about the use of Chi square for model evaluation in CFAs with large sample sizes, and that we base our inferences on other goodness of fit indices in our analyses. While our results except for the significant Chi square statistics corroborate that ESSI provides a single measure of social support across emotional, instrumental, informational and appraisal support, it is important to notice that ESSI assesses what has been labeled “perceived social support”, i.e. an individual’s perception of availability of social support [50] rather than enacted support or properties of social networks. Our results support the unidimensionality of ESSI, suggesting that different forms of support assessed by ESSI items can be assumed to indicate a single general degree of availability of social support. To our knowledge, only one previous study has applied CFA to examine the structure of ESSI [51] and has arrived at the same conclusion about the unidimensionality of the measure although among a large clinical sample. Furthermore, our results regarding gender invariance of ESSI indicate that the measure performs similarly and comparably well for both refugee men and women, an important assumption for our further analyses.

Perhaps most importantly, our results reveal that availability of post-resettlement social support partially mediates the association between exposure to torture and PTSD. This pattern could be interpreted in two conceptually related but somehow different ways. First, interpreting the found mediation as an explanation of the effect of exposure [52] suggest that being exposed to torture not only directly leads to higher odds for developing PTSD, but also has a detrimental influence on the individual’s social interactions and social support, which in its turn further heightens the risk for development of PTSD. Such an explanation is in line with the suggested psychological and social effects of torture in terms of distrust for others as well as uprooting from previous social networks [53]. Second, given that our results indicate that the impact of torture on PTSD in part flows via lower social support, it can be seen as suggesting that enhancing post-trauma social support resources may reduce the effect of torture exposure on development of PTSD. The latter interpretation, thus, may be viewed as supporting the notion of social support as a protective factor [54] in the established linkage between exposure to torture and development of trauma [9] which also is evidently corroborated by our results. Our results are in line with the PTSD literature ascribing a protective role to social support [55, 56], mainly based on the stress buffering model [16] which suggests that supportive social networks enhance individuals’ coping with stressful life events to buffer against the development of stress-related psychopathology. In the same vain, uprooted social networks due to forced migration may lead to disruption of sources of social support that may further aggravate the impact of torture exposure on PTSD. In addition to providing enhanced coping resources, social support in form of co-ethnic ties has been suggested to contribute to mental health of Syrian refugees by maintaining a sense of identity, belonging and efficacy [57]. This may constitute another protective pathway of social support in the linkage between severe trauma exposure of torture and PTSD.

Alternatively, the results of our study may be explained by viewing PTSD as an eroding influence on post-trauma social support [58]. This would render social support as an outcome rather than a mediator in the examined pattern of associations. Our sensitivity analyses, however, indicate that in the specific population of refugees and the context of torture’s effect on PTSD, modeling social support as a mediator is somehow preferable. However, these competing explanatory models, as well as a bi-directional model of social support and PTSD [59] among refugees exposed to torture, should be further examined by means of longitudinal studies.

Our results regarding the potential role of gender indicated that no moderation could be observed. Thus, our findings reveal that gender does not influence the mediatory function of social support in the association between torture and PTSD. Given the widely recognized gender and sex differences in the rates of PTSD [33, 60], the suggested gender differences in use and structure of social support [47], and the role of gender in the social sequelae of migration (in the domains of family, social network and availability of social resources) [61], a difference in the magnitude of and/or the function of social support in relation to mental health consequences of torture victimization could be expected. The findings that social support function similarly for both refugee women and men, however, is in line with our previous findings indicating a lack of pronounced gender difference in the rates of PTSD [11] and low social support [25] in the very same population. These results may be explained by the process of forced migration, which through disruption of pre-migratory social networks, a natural consequence of the uprooting of their social worlds [22, 23], limits the access to larger social networks outside the closed relationships, and thus, overrides the suggested differences in gendered social networks [62], rendering the use of social support for both refugee men and women in the early stages of migration more similar.

In all, our results lend robust empirical support to the role of enhanced social support as a potential protective factor for refugees who have survived torture. Our results also display that torture, as a grave violation of human rights and with severe health consequences for the individual, has detrimental effects of the victims’ social interaction and resources for social support. Furthermore, we show that both genders are affected similarly concerning effect of social support on PTSD and may benefit alike of efforts to enhance post-resettlement social support resources.

### Strengths and limitations

A unique strength of the study is that it is based on a large random sample of Syrian refugees selected from a comprehensive sample frame, providing means of analyses that are not possible in smaller, non-random selected clinical samples. However, the rather large non-response rate is a limitation of the study. Considering that refugees constitute a hard to reach population, as witnessed by the large number of studies addressing mental health consequences of torture in small clinical samples, our study may still provide substantially more reliable results. Moreover, although non-response rates may render finite population characteristics such as prevalence estimates biased, within-subject associations analyses such as those in the present study have been suggested to be less prone to such bias [63–65]. Our sensitivity analyses comparing the results of the main analyses based both on unweighted and available weighted data further corroborated that the results of the present study would remain unaltered by weighting the data sets.

Another strength of our study is the use of validated, standardized instruments for assessment of PTSD and Social support. However, a limitation of this study is the use of self-report data in the assessment of PTSD. Although clinical interviews may hold stronger validity, the possibility of conducting such interviews in large-scale studies and among hard to reach populations are inevitably constrained for logistic and practical reasons. Moreover, assessment of torture by a single self-report item may be considered a limitation. This approach may be justified by the difficulties inherent in assessing objective information on a sensitive issue such as torture exposure in large-scale studies [37]. The instrument to measure social support does not capture all issues related to social support e.g. information on the size of social network or where the support comes from are not included, which might be seen as a limitation. Furthermore, an important limitation in our study is that data on sex is used as a proxy for gender [46]. Although we are aware of the conceptual and methodological discrepancies between the concepts of sex and gender, logistical constraints limited our study’s ability to include in-depth analyses that would clarify potential empirical differences between these concepts. It is also important to note that the participants in this study were all granted residency in Sweden, the results may not be generalizable to asylum seekers who are waiting refugee status determination.

Finally, although a strength of the study is that statistical analyses represent advance modeling methods for assessment of mediation and moderated mediation in which all the estimates and several structural paths are acquired simultaneously, an important limitation is the lack of sufficient pre-migratory confounders such as more fine-grained sociodemographic variables e.g. socio-economic position. Perhaps more importantly, the design of the study is cross-sectional and, thus, insufficient for providing causal information. However, as self-reports on exposure refers to a previous experience, we assume that the links involving self-report on torture can be viewed as sufficiently causal. It is important also to note that despite inferences drawn from our sensitivity analyses, the directionality of the links between social support and PTSD cannot be viewed as empirically established due to the cross-sectional design of the study.

### Future research

In regard to future research, directionality and potential reciprocity of the impacts of social support and PTSD among refugees needs to be corroborated by longitudinal cohort design studies. In depth examinations of social support components and social networks properties, and the mechanisms by which these facets of social support may enhance health and resiliency among trauma-afflicted refugee are among other directions for future research that our results particularly imply.

## Conclusions

The results of this study provide unique preliminary evidence for the importance of supportive relations and ties for mental health of refugees who have been victims of severe trauma and human rights violations. Given the strained mid- and long-term specialized health care systems of many refugee-receiving countries [66], and the high prevalence levels of mental ill health among trauma-afflicted refugees, it can be concluded that facilitating, mobilizing and enhancing refugees’ social support should be considered a public health policy agenda. Conversely, it should be noted that policies that constrain and impede access to close supportive relationships, such as policies that lead to family fragmentation and limit family reunifications risk depriving trauma-afflicted refugee populations from important means and resources for recovery and health.

## Data Availability

Under Swedish law and ethical approval, individual level data of this kind cannot be publicly available. Individual level data can be made available on reasonable request as long as it is in line with Swedish law and ethical approvals.

## Abbreviations

BIC: Bayesian information criterion
CFA: Confirmatory factor analysis
CFI: Comparative fit index
CI: Confidence interval
df: Degrees of freedom
DSM-IV: Diagnostic and statistical manual of mental disorders, 4th edition
ESSI: ENRICHD Social Support Inventory
HTQ: Harvard trauma questionnaire
MI: Modification indices
ML: Maximum likelihood
MLR: Maximum likelihood estimation and robust standard errors
OR: Odds ratio
PTSD: Post-traumatic stress disorder
RMSEA: Root mean squared error of approximation
RTHC: The refugee trauma history checklist
_S-B_χ2: Satorra–Bentler scaled chi-square
SD: Standard deviation
SEM: Structural equation modeling
SRMR: Standardized root mean square residual
TPR: Total population register
